# Amputation of the Leg below the Knee

**Published:** 1838-02-14

**Authors:** 


*Amputation of the Leg below the Knee.*

David Lewis, a black, aged 28 years, was admitted into the hospital on the 14th of October, 1837, for caries of the ankle joint, from a sprain which occurred two years previous. From the date of his admission, efforts had been made to save the limb, by the use of the carved splint, the exhibition of tonics, and absolute rest,—but without success. Amputation having been determined upon, Dr. Harris performed the operation, January 24th, present Drs. Randolph and Norris. He made a circular horizontal incision, four inches below the knee-joint. The bone was first sawed transversely, and the spine of the tibia afterwards taken off obliquely. The edges of the wound were drawn together so as to leave the opening in a vertical direction.

*February* 8th. To-day the principal ligature came away, the others having been removed yesterday. The man is doing well—the wound nearly closed, and he has not had a bad symptom since the operation.

An operation was performed for varicose veins, on the 2nd inst. The result will be reported in No. 5.

